# Variation in subsurface thermal characteristics of microrefuges used by range core and peripheral populations of the American pika (*Ochotona princeps*)

**DOI:** 10.1002/ece3.2763

**Published:** 2017-02-07

**Authors:** Thomas J. Rodhouse, Matthew Hovland, Mackenzie R. Jeffress

**Affiliations:** ^1^ US National Park ServiceBendORUSA; ^2^ Department of Fish and WildlifeUniversity of IdahoMoscowIDUSA; ^3^ Nevada Department of WildlifeElkoNVUSA; ^4^ Present address: Department of Animal and Rangeland SciencesOregon State University112 Withycombe HallCorvallisOR97331USA

**Keywords:** American pika, climate change, microclimate, microrefuge, National Parks, *Ochotona princeps*, range periphery, temperature

## Abstract

Microrefuges provide microclimates decoupled from inhospitable regional climate regimes that enable range‐peripheral populations to persist and are important to cold‐adapted species in an era of accelerated climate change. However, identifying and describing the thermal characteristics of microrefuge habitats is challenging, particularly for mobile organisms in cryptic, patchy habitats. We examined variation in subsurface thermal conditions of microrefuge habitats among different rock substrate types used by the American pika (*Ochotona princeps*), a climate‐sensitive, rock‐dwelling Lagomorph. We compared subsurface temperatures in talus and lava substrates in pika survey sites in two US national park units; one park study area on the range periphery and the other in the range core. We deployed paired sensors to examine within‐site temperature variation. We hypothesized that subsurface temperatures within occupied sites and structurally complex substrates would be cooler in summer and warmer in winter than unoccupied and less complex sites. Although within‐site variability was high, with correlations between paired sensors as low as 47%, we found compelling evidence that pikas occupy microrefuge habitats where subsurface conditions provide more thermal stability than in unoccupied microhabitats. The percentage of days in which microhabitat temperatures were between −2.5 and 25.5°C was significantly higher in occupied sites. Interestingly, thermal conditions were substantially more stable (*p* < .05) in the lava substrate type identified to be preferentially used by pikas (pahoehoe vs. a'a) in a previous study. Our study and others suggest that thermal stability appears to be the defining characteristic of subsurface microrefuges used by American pikas and is a likely explanation for enigmatic population persistence at the range periphery. Our study exemplifies an integrated approach for studying complex microhabitat conditions, paired with site use surveys and contextualized with information about gene flow provided by complementary studies.

## 1 Introduction

The importance of microhabitats in ecology has increased in recent years (e.g., Ashcroft et al., 2012; Hannah et al., 2014; Potter et al., 2013; Scheffers et al., 2014), due in part to concerns about accelerating climatic changes that adversely affect species adapted to colder climates than currently exist within their historic range (i.e., glacial relicts, Williams et al., 2015). Microhabitats offer alternative resources, which can include microclimates, to those found in the surrounding ecosystem. Thermal microrefuges are microhabitats that may facilitate persistence of populations in peripheral regions of species’ ranges where climatic stressors exceed physiological limits (i.e., microrefugia; Ashcroft, 2010; Dobrowski, 2010; Keppel et al., 2012; Rull, 2009). Organisms adapted to cooler climates have utilized thermal microrefuges in the current interglacial period as a means to survive in areas where regional climates and resources have become unsuitable due to changing environmental conditions (Hampe & Jump, 2011; Scherrer & Körner, 2011). Although peripheral populations are often considered to be more prone to extirpation, they may also be more resilient than range core populations because of the evolutionary exposure to environmental conditions that are more stressful than those in the range core. Populations persisting on range peripheries may provide sources of genetic diversity, introducing their adaptations to these higher rates of environmental stress to core population gene pools facing fewer selective pressures (Kark et al., 2005; Sexton et al., 2009).

To better understand and further define microrefuge, habitats enables us to model, map, and conserve areas that may harbor the most resilient and even the sole remaining populations of a species which evolved fitness‐conferring traits following periods of glaciation. These peripheral, microrefuge‐dependent populations are often underrepresented in species distribution models as climate data are usually based on regional climates rather than the often decoupled microclimates (Mosblech, 2011; Potter et al., 2013; Randin et al., 2009; Scherrer et al., 2011). This underrepresentation is in part due to the conceptual and practical limits of knowledge about how best to identify and describe microrefuges, especially for mobile organisms that occupy cryptic, inaccessible microhabitats. Recent studies have attempted to define thermal microrefuges by modeling the factors which may contribute to thermal heterogeneity (Potter et al., 2013) and buffering (Shi et al., 2014), and in some cases even detailing the processes of airflow that create microclimates (Daly et al., 2010; Millar et al., 2014). However, as a species‐specific concept, microrefuges cannot be identified and described as suitable using only broad ecological characterizations. Rather, such characterizations must be integrated with observations made at biologically appropriate fine scales utilizing metrics relevant to the physiological requirements of the species concerned (Ashcroft et al., 2012; Keppel et al., 2012). In the case of mobile organisms, this should include verifying preferential ecological resource use (Turlure et al., 2009; : Hall et al., 2016).

**Figure 1:**
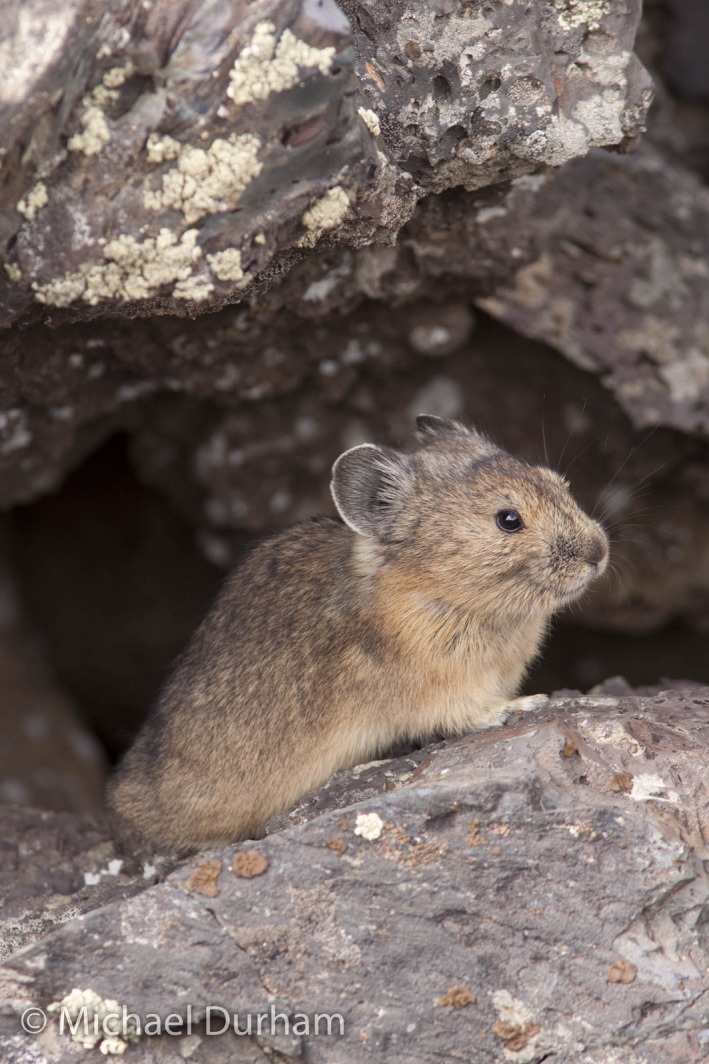
American pika (*Ochotona princeps*) in pahoehoe lava at the Craters of the Moon study site. Photograph courtesy of Michael Durham (www.durmphoto.com), reproduced with permission

In this article, we describe an investigation into the thermal characteristics of subsurface microrefuge habitats used by the American pika (*Ochotona princeps*), a climate‐sensitive rock‐dwelling Lagomorph (Figure 1). This species provides for an interesting conceptual challenge because of its reliance on highly complex and patchily distributed subsurface habitats (Moilanen et al., 1998; Smith, 1974; Smith & Nagy, 2015). The existence of thermal microrefuges has been offered as a hypothesis for persistence of the American pika in low‐elevation range peripheries (Hall et al., 2016; Jeffress et al., 2013; Millar & Westfall, 2010; Millar et al., 2016; Rodhouse et al., 2010; Shinderman, 2015; Varner & Dearing, 2014), although this has not been widely tested. We do so by comparing subsurface temperature measurements in occupied and unoccupied sites among different rock substrate types in two US National Park study areas—one located on the range periphery and a second in the range core. Substrate types included montane talus and two types of lava, a'a and pahoehoe (Kuntz et al., 2007), where previous research showed that pika site occupancy probabilities were ~10 times greater in pahoehoe than a'a lava (Rodhouse et al., 2010). We hypothesized that (1) subsurface microclimates in occupied sites would be colder in the summer and warmer in the winter than in unoccupied sites, (2) substrates of increasing structural complexity (i.e., size and depth of boulders) would provide increasing levels of thermally stable microclimates, and (3) microclimate thermal stability would differ between a'a and pahoehoe lava substrates, providing a mechanistic explanation for the strong difference in occupancy probabilities observed by Rodhouse et al. (2010) and for range periphery persistence, more generally. We paired temperature measurements with site occupancy surveys in order to distinguish between used and unused sites, and we deployed pairs of sensors at each survey site in order to investigate temperature variation within microsites.

## 2 Materials and Methods

### 2.1 Study organism

The American pika is an obligate denizen of subsurface interstices of talus fields and lava flows in the mountainous areas of western North American. These habitats provide structurally complex substrates which contribute to the formation of topographically mediated microclimates. Recent studies utilizing temperature sensors within pika habitats have revealed the existence of subsurface microclimates decoupled from regional air temperatures (e.g., Hall et al., 2016; Henry et al., 2012; Millar et al., 2014, 2016; Varner & Dearing, 2014; Wilkening et al., 2011). The species is sensitive to heat loading during summer foraging bouts, with experimental exposure to temperatures in excess of 25.5°C for even brief periods having been shown to cause stress and death (Smith, 1974). In order to avoid daytime extreme temperatures, pikas remain in subsurface talus and lava interstitial spaces (Figure 1) and utilize crepuscular foraging habits to limit daytime activity. Pikas residing at low elevations tend to be less active during the day and exhibit altered space use and foraging patterns than those at higher elevations (Henry et al., 2012; Smith, 1974; Varner et al., 2016). Declining mountain snowpack and exposure to acute winter cold temperatures resulting from accelerated global change has also been advanced as an additional source of stress and population decline for American pika (Beever et al., 2010; Erb et al., 2011; Guralnick et al., 2011).

There is evidence that American pika physiological adaptations to colder climates have become limitations during current periods of warming. It is believed that since the last glacial maximum, increasing temperatures have been a contributing factor to range contractions in the Great Basin and Sierra Nevada mountains (Beever et al., 2003; Galbreath et al., 2009; Wilkening et al., 2011). Species distribution models predict further contraction at low latitudes and elevations (Calkins et al., 2012; Galbreath et al., 2009; Stewart et al., 2015). However, the small body size (Figure 1), low thermal tolerance, use of structurally complex habitats, and behavioral adaptations of the American pika suggest a scenario of persistence under microrefugia population models (Mosblech, 2011). Small mobile organisms such as the American pika have greater access to thermal microhabitats than larger or more sessile organisms (Potter et al., 2013) and can decrease energetic costs of thermoregulation with behavioral adaptations to microhabitat availability (Sears & Angilletta, 2015; Varner et al., 2016). Indeed, scenarios of persistence in the face of climate change have emerged from modeled projections of American pika distributions when local‐scale factors including topographic position and habitat patch configuration are accounted for (Schwalm et al., 2016).

### 2.2 Study areas

**Figure 2:**
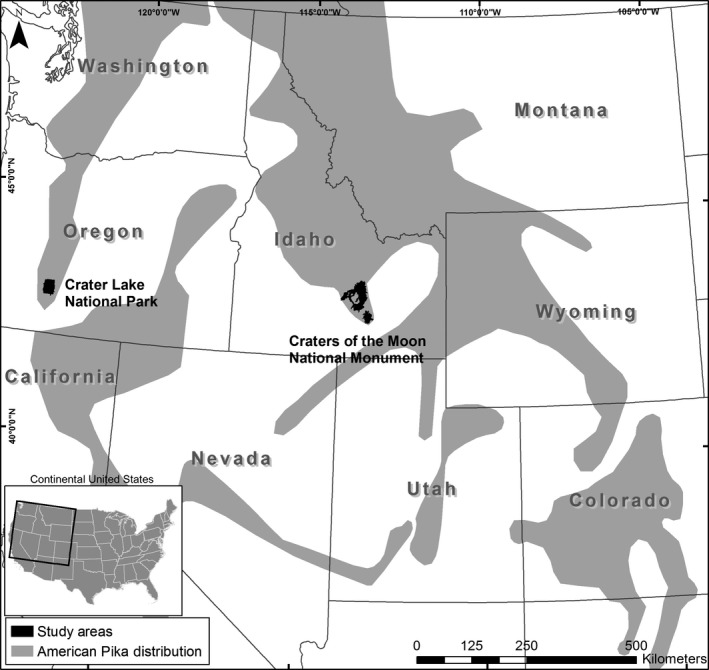
Map of the two US National Park study areas and a highly generalized range map of the American pika published by the International Union for Conservation of Nature and Natural Resources (Beever & Smith, 2011). Revisions to this range map are forthcoming (E. Beever and A. Smith, personal communication). The park study areas are within the ranges of two distinct American pika phylogenetic lineages, *O. p. fenisex* and *O. p. princeps* (Beever & Smith, 2011; Galbreath et al., 2010)

We recorded fine‐scale temperature and site occupancy patterns during 2010–2013 in two US National Parks: Craters of the Moon National Monument and Preserve (hereafter Craters of the Moon), located in the Snake River Plain of southeast Idaho (43°35′N, 113°50′W), and Crater Lake National Park (hereafter Crater Lake), located in southwestern Oregon in the Cascade Range (42°57′N, 122°6′W; Figure 2). Craters of the Moon consist of approximately 1,025 km^2^ of basalt lava flows (Figure 2), which extend along an elevational gradient from 1,511 to 1,833 m. The climate is semi‐arid, with a 30‐year average annual precipitation of 390 mm (Western Regional Climate Center [WRCC] station #102260). The summer drought period occurs from June through September, with July maximum temperatures averaging 29°C. Thirty‐year average snow depth peaks in February at 635 mm, although a weather station (WRCC #105972) ~70 km south of the study area recorded an average of only 25 mm, underscoring the elevation and associated climatic gradient of the study area. The average minimum temperature in January is −12°C.

**Figure 3:**
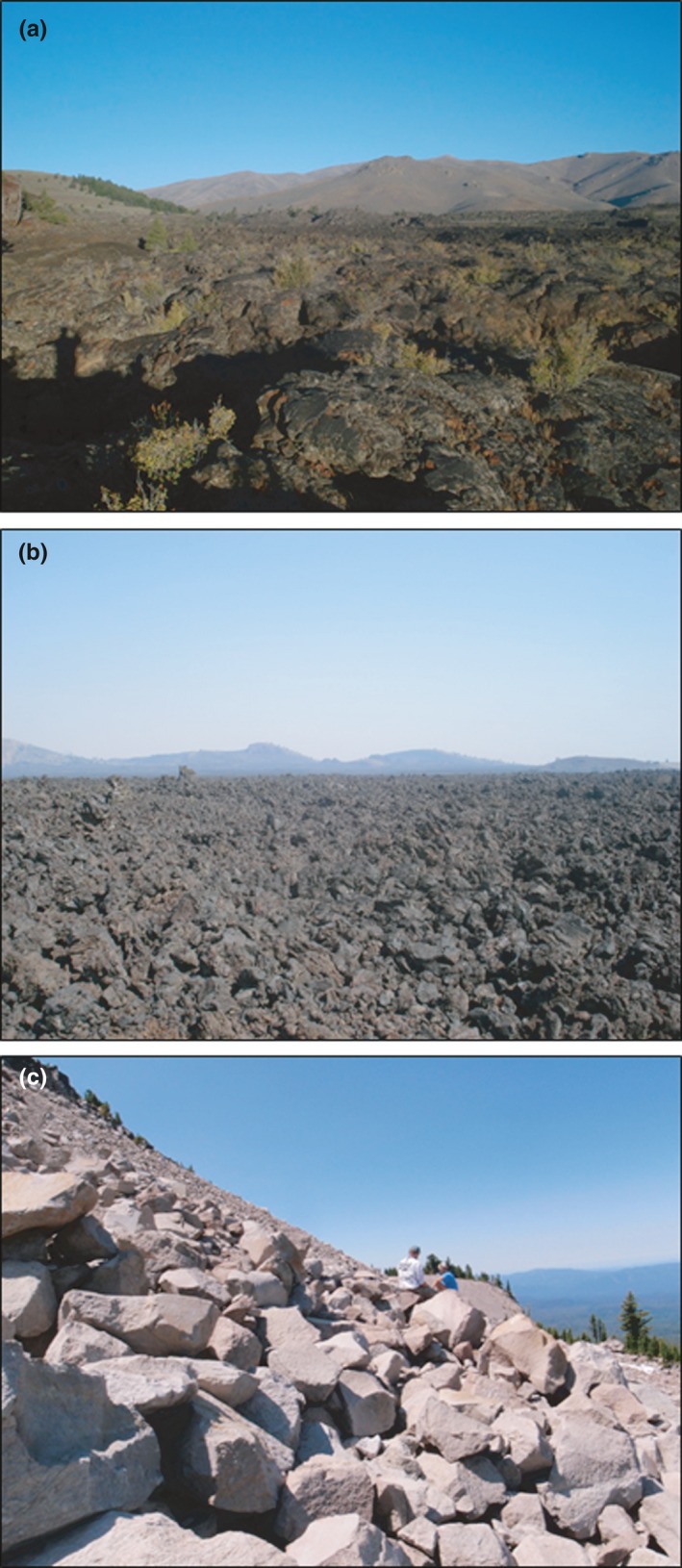
Photographs of (a) pahoehoe lava and (b) a'a lava at Craters of the Moon, and (c) a boulder‐field substrate in Crater Lake

Importantly, Craters of the Moon occurs on an interior range periphery for the American pika (Smith & Weston, 1990; Galbreath et al., 2010; Rodhouse et al., 2010; Figure 2) and provides marginal habitat conditions (e.g., low, hot, and dry) relative to current understanding of the species’ needs (Hafner, 1993; Jeffress et al., 2013; Rodhouse et al., 2010). In contrast, Crater Lake is positioned well within the species (albeit patchy) range core (Smith & Weston, 1990; Figure 2) and is considerably cooler than Craters of the Moon, with a 30‐year average annual precipitation of 166 cm (WRRC #351946) and a relatively brief summer drought period during July and August. Thirty‐year maximum July temperatures for Crater Lake average 21°C. Thirty‐year average snow depth in Crater Lake peaks in March at ~3 m, while 30‐year average minimum temperature in January is −8°C. With these climatic conditions, the steep talus and boulder‐field slopes (Figure 3) surrounding the crater rim (the park being named after its centerpiece water‐filled volcanic caldera of the same name) and found on other ridges and peaks within the park provide more typical (e.g., Smith & Weston, 1990) montane pika habitat than is found in Craters of the Moon. Elevations in Crater Lake range from 1,701 to 2,530 m. The findings from pika site occupancy surveys indicate that the number of occupied sites is greater and less prone to turnover in Crater Lake than in Craters of the Moon (Jeffress et al., 2013; Rodhouse & Hovland, 2015). The proportions (*n *=* *103) of occupied sites in Craters of the Moon have fluctuated widely from as low as 0.07 to 0.21 over a 5‐yr period (2010–2014), whereas proportions (*n *=* *100) of occupied sites at Crater Lake consistently remained >0.50 over the same period (Rodhouse & Hovland, 2015).

### 2.3 Study design

**Figure 4:**
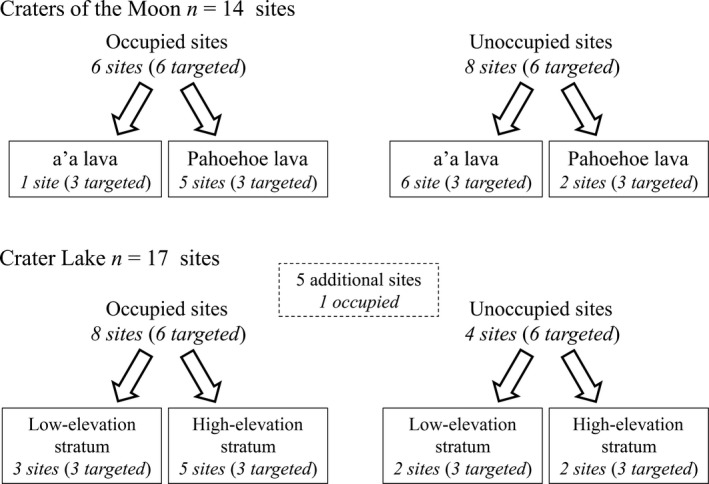
American pika microrefuge experimental design schematic, reflecting a priori hypotheses and targeted and achieved sample sizes. All sites except the five additional sites at Crater Lake were instrumented with two sensors and were placed at randomly located long‐term pika site occupancy monitoring plots. The five additional sites were placed in nonmonitored sites in the caldera where fresh (one site) and old (four sites) pika sign had been found. Discrepancies between targeted and achieved sample sizes reflect the challenge of prospective (as opposed to retrospective) experimental designs in field ecology. In particular, we sought out but were unable to find enough actively occupied a'a lava sites in Craters of the Moon, underscoring the preferential use patterns exhibited by pikas at Craters of the Moon for pahoehoe lava substrate

Temperature sensors (Onset Hobo water temp v2^1^) were deployed in September 2010 at 14 of the ~100 randomly selected pika monitoring sites established at Craters of the Moon and at 12 monitoring sites established at Crater Lake (Figure 4). Sensors were also included in five additional nonrandomly selected sites at Crater Lake where pika site occupancy surveys had also been conducted but where limited access (caldera lakeshore and island) precluded regular monitoring, resulting in a total of *n *=* *31 sites (Figure 4). All but five sites received two sensors to examine within‐site variability. Most sensors (48 of 57) logged data for >1,000 days. Seven loggers were removed after 365 days, and two sensors were removed after 734 days due to study logistics.

Long‐term monitoring in the study area was established by Jeffress et al. (2011) following methods introduced by Rodhouse et al. (2010). Survey sites were 24‐m‐diameter circles, with plot centers determined from a spatially balanced random sample from within sampling frames constructed in a GIS as mapped lava and talus potential habitat. The dimensions of plots correspond well to pika territory sizes (Smith & Nagy, 2015; Smith & Weston, 1990; Varner et al., 2016). Surveys were conducted each summer during 2010–2013, and sites where fresh active sign were found at least once during those four surveys was considered occupied for the purposes of this study. Site occupancy status established in summer 2010 was used as the design factor that guided temperature logger deployment to occupied and unoccupied sites (Figure 4). Although imperfect detection is a potential confounding factor, we previously estimated detection probabilities >0.90 for pikas using the same survey protocol in Craters of the Moon (Rodhouse et al., 2010) and assumed that probability of detecting site use at least once over the four survey periods would be as high. At occupied sites, sensors were placed in the crevices nearest to the plot center with active sign. Second sensors were placed nearby within the same 12‐m‐radius plot in similar crevice type and depth conditions and the distance to nearest active sign was recorded. At unoccupied sites, sensors were placed in the nearest suitable crevices to the survey plot center.

At Crater Lake, all 17 sites consisted of “typical” (for the American pika, sensu Smith & Weston, 1990) montane talus (which in this study area is actually derived from weathered high‐silica rhyodacite block lava (Bacon & Lanphere, 1990)) substrate (Figure 2). At Craters of the Moon, we selected seven sites in a'a lava substrate (Figure 3) and seven sites in pahoehoe lava substrate (Figure 3; these Hawaiian names for basaltic lava flows are standard usage among geologists (e.g., Peterson & Tilling, 1980; Kuntz et al., 2007)), following surface land cover classification and mapping by Bell et al. (2009) and as verified during field surveys.

### 2.4 Statistical analyses

Hourly temperature data were compiled in Hoboware as maximum, minimum, and average daily temperatures, and exported for analysis. These data are archived and publicly available online (https://irma.nps.gov/DataStore/Reference/Profile/2237809). We evaluated the correlation between hourly time series from paired sensors using Kendall's τ, a rank‐based concordance index or correlation coefficient (McLeod, 2011), computed in the R statistical environment (R Core Team 2014). We subtracted paired time series to calculate the mean, maximum, and minimum discrepancies. The number of days exceeding 25.5°C was calculated using both maximum (acute stress) and average (chronic stress) daily temperatures as a way to assess site suitability relative to the apparent upper limit of pika thermal tolerance. The number of days in which temperatures dropped below −2.5°C was also calculated as a proxy for snowpack. Millar et al. (2014) observed similar temperature limits in eastern Sierra talus slopes with heavy snow pack (−2.0°C), and we assumed that sites below this limit lacked thermal insulation, and would therefore expose pikas to cold stress. The number of days within the 25.5 and −2.5°C limits was also calculated. Finally, the number of days of snow cover was estimated by proxy using a 1 to −2.5°C buffer. We converted the raw counts of days that met the respective temperature thresholds to percentage of days in which temperatures were recorded because of variation in the number of recording days among sites.

We adopted a model‐based analysis of covariance (ANCOVA) approach for testing hypotheses, a familiar regression strategy well‐suited to handle the study design imbalance and account for both the important study design factors of interest and the multiple environmental site attributes measured as numeric continuous (as opposed to factors) variables (Huitema, 2011). We compiled box‐and‐whisker plots (boxplots) and developed regression models with data from the 57 sensors to test our three hypotheses with the following response variables: (1) % days with maximum temperatures >25.5°C (acute hot days), (2) % of days with minimum temperatures <−2.5°C (acute cold days), (3) % of days in which the temperatures remained within −2.5 and 25.5°C (stable days), (4) % of days in which temperatures remained within −2.5 and 1°C (snow days), (5) % of days in which average temperatures >25.5°C (chronic hot days), and (6) % of days in which average temperatures <−2.5°C (chronic cold days). These variables were mean‐centered and modeled with an identity link using generalized least squares (GLS) with first‐order autoregressive error (i.e., AR‐1 model) using site as a grouping factor to account for serial correlation between the paired sensors (Venables & Ripley, 2002; Zuur et al., 2009). Given the structure of our dataset, this generalized to using a compound symmetric covariance structure with a single off‐diagonal correlation parameter *φ*, essentially accounting for pseudoreplication and effective degrees of freedom. We used the *nlme* package (Pinheiro, Bates, DebRoy, Sarkar, & R Core Team, 2014) in R to fit models. We assessed sensitivity of results to design structure by fitting a model without the data from the five nonrandom sites in Crater Lake. We assessed sensitivity of results to model structure by fitting a model without AR‐1 error to a dataset with only one sensor per site. We also fit a binomial regression model (as with GLS but with a binomial logit link and a different optimization routine) to noncentered response data (i.e., proportions), with and without a random effect for site (Stroup, 2014) using the *lme4* package (Bates et al., 2014) in R. We found no differences in interpretation (i.e., the sign and relative magnitude of estimated coefficients did not vary substantially, although standard errors were smaller with the binomial model; results not shown) even when only one sensor per site was used; random‐effects models did not converge well due to having only two sensors per site. Correlation among sensors was surprisingly low in some sites (see Results), and the estimated correlation parameter *φ* was also small, ranging from 0.04 to 0.28.

We accounted for site environmental variation by including covariates for topography, elevation, forb cover, and sensor depth. Topography and elevation were estimated using 10‐m digital elevation models. Topography was calculated as sin(slope) * cos(aspect), which created a numeric variable ranging from −1 to 1, where steep north slopes approach 1 and steep south slopes approach −1 (Jeffress et al., 2013). We constructed elevation as a continuous variable rather than as the factor used for the design (Figure 4) to better incorporate the range of elevations represented from all 31 sites. Forb cover was estimated visually following the occupancy survey protocol developed by Rodhouse et al. (2010) and Jeffress et al. (2011). Covariates were centered on zero, and in the case of elevation and sensor depth, standardized to improve computation and interpretability of estimated coefficients (specifically the intercept; Schielzeth, 2010). Mean elevation was 1,912 ± 222 m. Mean sensor depth was 60 ± 24 cm.

To test hypotheses, we added indicator variables into models for three design factors (see Figure 4). Occupancy status was constructed as a two‐level factor (occupied and unoccupied), and substrate type (a'a, pahoehoe, and talus) and site structural complexity (of rock substrate) were constructed as three‐level factors. Site complexity was evaluated in the field during surveys using three categories, following methods used successfully by Rodhouse et al. (2010) and Shinderman (2015) to distinguish pika site occupancy probabilities among sites: low complexity (rank 1), moderate complexity (rank 2), and high complexity (rank 3). The resulting model structure used for each of the six response variables was **Y**_*i*_ **~ *N*(**Xβ**,** V**_*i*_), where **V**_*i*_ = cov(σ^2^, *φ*), and **X** = [elevation, topography, forb cover, sensor depth, complexity rank_*j*−1_, substrate_*j*−1_, occupancy status_*k*−1_, and occupancy_*k*−1_ × substrate_*j*−1_] with *j* and *k* indexing factor levels. Pearson's ρ and Kendall's τ (for factors) correlation coefficients among all model inputs were small (<0.2) except between forb cover and elevation, which was 0.6.

## 3 Results

### 3.1 Fine‐scale (within‐site) microclimate variation

We discovered substantial discrepancies between paired sensors, with concordances (τ) as low as 0.47 and discrepancies as large as ±14°C in some sites (Table 1). Overall mean concordance was higher at Craters of the Moon (0.87) than at Crater Lake (0.71). Interestingly, microsites with large maximum temperature discrepancies did not also consistently record large minimum temperature discrepancies, and vice versa (Table 1). This revealed that some microsites were hotter but not colder than a neighbor and that others were colder but not hotter than a neighbor. In at least two sites (Crater Lake 36 and 192; Table 1), discrepancies were equally large for both cold and hot ranges, and concordance was very low (0.47 and 0.58; Table 1). In some sites (e.g., Crater Lake 216 and 726; Table 1), discrepancies were relatively small in both directions, yet still exhibited relatively low concordance (0.73 and 0.75; Table 1). Consistent with these findings were the small *φ* values estimated from models as well (0.04–0.28).

**Table 1:** Mean daily temperature discrepancies (mean, minimum, and maximum) and Kendall's τ concordance, between pairs of sensors placed within pika site occupancy survey sites in two US National Park study areas during 2010–2013

Park	Site	Kendall's τ	Mean	Min	Max
CRLA	036	0.58	−0.33	−12.78	14.47
CRLA	096	0.86	−1.29	−8.56	1.79
CRLA	192	0.47	−0.41	−14.27	13.17
CRLA	216	0.73	0.17	−3.30	2.66
CRLA	232	0.79	0.21	−1.89	5.68
CRLA	283	0.79	0.07	−6.05	12.00
CRLA	519	0.50	3.04	−5.93	18.82
CRLA	549	0.72	−0.52	−2.86	9.77
CRLA	552	0.88	−0.19	−4.05	2.67
CRLA	585	0.76	−0.28	−6.49	5.40
CRLA	626	0.70	1.57	−8.01	16.99
CRLA	726	0.75	−0.27	−3.37	2.44
CRMO	003	0.84	3.70	−0.87	15.31
CRMO	081	0.89	0.23	−5.93	5.86
CRMO	083	0.92	0.57	−4.48	8.14
CRMO	085	0.92	−0.84	−14.20	3.04
CRMO	086	0.88	−0.60	−7.83	10.35
CRMO	096	0.90	−1.88	−6.06	3.91
CRMO	100	0.83	0.10	−4.48	11.59
CRMO	101	0.86	−1.90	−14.46	6.25
CRMO	241	0.80	0.22	−14.77	8.09
CRMO	242	0.90	−1.34	−6.51	2.80
CRMO	243	0.89	−1.27	−9.37	3.35
CRMO	245	0.89	−1.19	−9.38	6.35
CRMO	246	0.87	0.17	−6.93	7.27
CRMO	248	0.92	0.89	−2.29	7.53

### 3.2 Microclimate differences among sites

Across all six response models (Table 2), the influences of the site environmental attributes (elevation, topography, forb cover, and sensor depth) on % of days meeting respective microsite temperature thresholds were generally small, with estimated coefficients <0.1 and *p*‐values >.10. The exception to this pattern was with topography, which had relatively large estimated effect sizes and small *p*‐values (<.05) in the two (acute and chronic) % cold days models (Table 2). This suggests, intuitively, given the Northern Hemisphere and mid‐latitude location of the study, that subsurface microsites on steeper, north‐facing slopes were consistently (on average) colder. Counterintuitively, microsite temperatures did not consistently differ along elevation and sensor depth gradients, nor was there any apparent relationship between site forb cover and microsite temperatures.

**Table 2:** Regression coefficient estimates, standard errors, and *p*‐values from six ANCOVA regression models testing hypotheses that, after controlling for variation in habitat conditions and sensor depth, subsurface microclimates differ significantly among pika‐occupied and pika‐unoccupied lava and talus substrate types. Data from all sensors were pooled for this analysis, and a first‐order autoregressive error structure was included in model structure to account for correlation between sensors paired within sites. “Bold‐font *p*‐values indicate statistical significance at alpha = 0.05

Parameter	% Stable days			% Snow days		
	Estimate	*SE*	*p*‐Value	Estimate	*SE*	*p*‐Value
Unoccupied Aa (Intercept)	−0.27	0.07	**.01**	−0.19	0.07	**.01**
Elevation	−0.01	0.03	.67	0.03	0.03	.32
Topography	−0.08	0.09	.39	0.01	0.08	.95
Forb Cover	−0.01	0.01	.85	0.01	0.01	.47
Sensor Depth	0.01	0.01	.28	−0.01	0.01	.44
Complexity Rank 2	0.01	0.04	.89	0.06	0.03	.13
Complexity Rank 3	0.05	0.04	.25	0.07	0.04	.09
Pahoehoe	0.15	0.06	**.02**	0.05	0.06	.36
Talus	0.28	0.07	**.01**	0.29	0.07	**.01**
Occupied	0.22	0.09	**.02**	0.19	0.08	**.02**
Pahoehoe × Occupied	−0.24	0.11	**.04**	−0.22	0.10	**.04**
Talus × Occupied	−0.14	0.10	.16	−0.08	0.09	.37

	% Acute hot days			% Acute cold days		
	Estimate	*SE*	*p*‐Value	Estimate	*SE*	*p*‐Value
Unoccupied Aa (Intercept)	0.14	0.05	**.01**	**0.12**	**0.06**	**.05**
Elevation	0.02	0.02	.34	−0.01	0.02	.82
Topography	−0.10	0.06	.11	0.18	0.07	**.02**
Forb Cover	−0.01	0.01	.16	0.02	0.01	.15
Sensor Depth	−0.01	0.01	.06	0.01	0.01	.76
Complexity Rank 2	0.01	0.02	.61	−0.01	0.03	.55
Complexity Rank 3	−0.01	0.03	.65	−0.03	0.03	.29
Pahoehoe	0.02	0.04	.58	**−0.18**	**0.05**	**.01**
Talus	−0.13	0.04	**.01**	**−0.14**	**0.05**	**.01**
Occupied	−0.05	0.06	.40	**−0.17**	**0.07**	**.02**
Pahoehoe × Occupied	−0.01	0.07	.84	**0.26**	**0.09**	**.01**
Talus × Occupied	0.03	0.06	.56	0.10	0.08	.20

	% Chronic hot days			% Chronic cold days		
	Estimate	*SE*	*p*‐Value	Estimate	*SE*	*p*‐Value
Unoccupied Aa (Intercept)	0.03	0.01	**.02**	0.21	0.05	**.01**
Elevation	0.01	0.01	.64	−0.01	0.02	.72
Topography	−0.01	0.02	.62	0.18	0.07	**.01**
Forb Cover	−0.01	0.01	.70	0.01	0.01	.16
Sensor Depth	0.01	0.01	.46	0.01	0.01	.82
Complexity Rank 2	−0.01	0.01	.98	−0.01	0.03	.58
Complexity Rank 3	−0.01	0.01	.24	−0.03	0.03	.31
Pahoehoe	−0.01	0.01	.99	−0.17	0.04	**.01**
Talus	−0.04	0.01	**.01**	−0.12	0.05	**.03**
Occupied	−0.04	0.02	**.04**	−0.18	0.06	**.01**
Pahoehoe × Occupied	0.01	0.02	.60	0.27	0.08	**.03**
Talus × Occupied	0.03	0.02	.07	0.13	0.07	.07

After accounting for this among‐site variation and low covariances between paired sites, we found compelling evidence ($\hat{\beta }$ > ±0.10 and *p* < .05) that sites occupied by pikas (i.e., actively used in at least one of the four study years) experienced greater percentage of stable (−2.5–25.5°C) subsurface temperatures, a higher percentage of snow‐covered days, and lower percentage of cold days (acute and chronic, <−2.5°C; Table 2). Evidence for differences in percentage of hot days between occupied and unoccupied sites was less clear, and only modestly (small coefficient and *p*‐value) apparent for chronic hot days (Table 2). Surprisingly, we found no evidence that microclimates differed among sites with different complexity rankings.

We found very clear evidence that microclimates differed among sites with different substrates and that there was significant interaction between substrate type and occupancy status.

**Figure 5:**
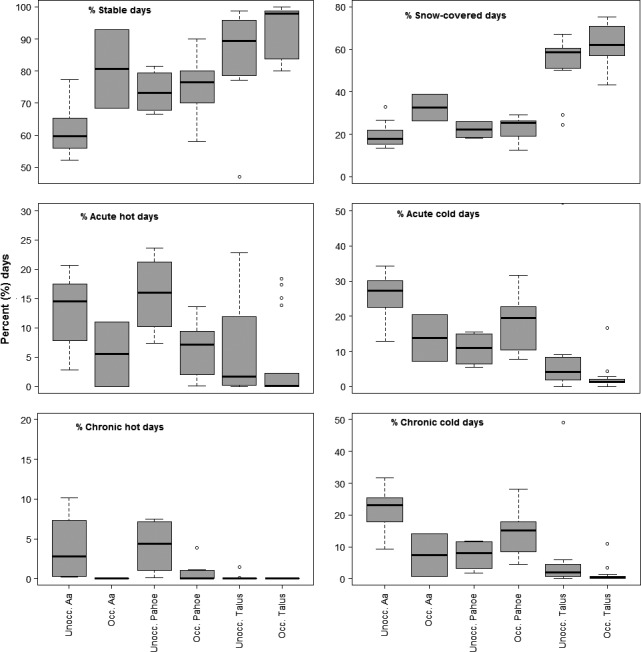
Boxplots showing variation in microclimate conditions of American pika occupancy monitoring sites among sensors placed in two lava substrate types, a'a (*n* = 7) and pahoehoe (*n* = 7), and montane talus (boulder fields; *n* = 17) in sites occupied (abbreviated here as “Occ.”) and unoccupied (“Unocc.”) by American pikas

**Figure 6:**
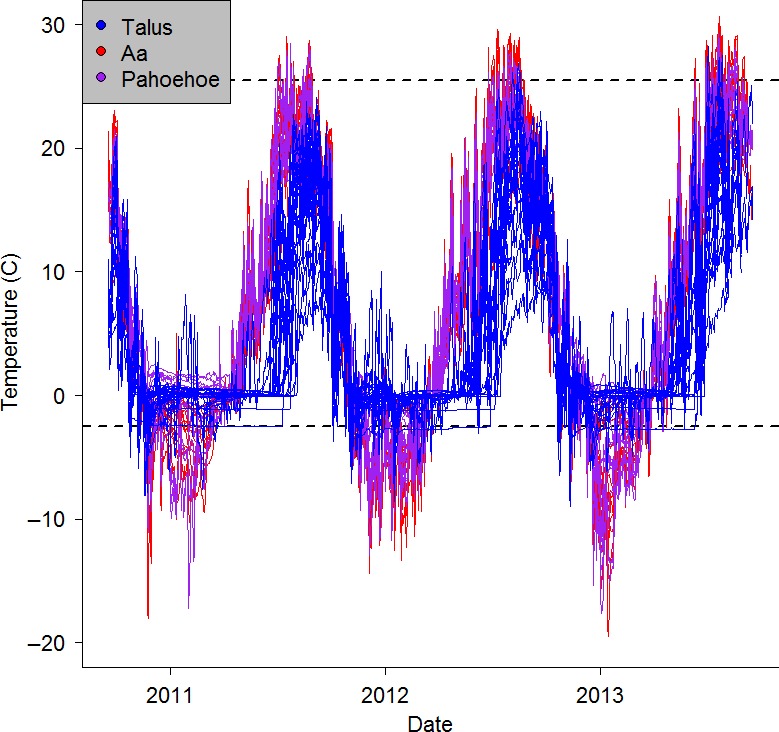
Plot of sensor temperature recordings obtained from 57 loggers placed in 31 American pika occupancy monitoring sites in two US National Parks. Temperature recordings are color‐coded by substrate type (talus, a'a, pahoehoe), and physiologically meaningful thermal extremes are represented by horizontal dotted lines at 25.5 and −2.5°C. Long‐duration snowpack is evident in some talus sites (flat ~0°C blue lines). Temperature extremes are evident in both lava substrates when compared with talus, and a'a (red lines) show greater extremes than pahoehoe (purple lines). Warming occurs considerably earlier each year in the lava substrates than in talus due to the much lower snowpack at Craters of the Moon

Most germane to our study objectives were the differences observed in Craters of the Moon between a'a and pahoehoe sites. The boxplots in Figure 5 illustrate how microclimates in unoccupied a'a were consistently less stable, less snow‐covered, hotter in summer, and colder in winter than the two sensors in the occupied a'a site. A similar pattern was observed between occupied and unoccupied pahoehoe sites, where occupied pahoehoe sites appear to have fewer hot days (Figure 5). Model‐based estimates of differences among group means, which reflects statistical uncertainty (including sample size and design imbalance), support the conclusion of significant difference, with relatively large coefficients and small *p*‐values for differences in means between unoccupied a'a (intercepts) and other groups (Table 2). In addition, pahoehoe sites were significantly more stable and experienced fewer extreme cold days in winter (Table 2). Significant differences were clear between talus sites at Crater Lake and a'a and pahoehoe groups in Craters of the Moon (Figure 5 and Table 2), reflecting the large regional climatic differences between the two study parks. Notably, sites in Crater Lake rarely exceeded chronic heat stress conditions (daily means >25.5°C; Figure 5) and were always much more stable, snow‐covered, and warmer in winter than in Craters of the Moon (Figures 5 and 6). Figure 6 shows the complete time series for all sensors, color‐coded by substrate type. The lower number of extreme temperature events and longer duration of snowpack in Crater Lake talus are apparent in Figure 6, as is a consistently earlier warming trend each summer in Craters of the Moon a'a and pahoehoe than in Crater Lake talus (delayed by later snowmelt). These park‐level differences account for the patterns in the coefficient estimates of substrate × occupancy status interaction terms, which in several models provided statistically significant mean adjustments to be made to occupied pahoehoe relative to talus (which exerted leverage on occupied group means).

## 4 Discussion

Our study provides compelling evidence that thermal stability is the defining characteristic of subsurface microrefuges for the rock‐dwelling American pika. Even after accounting for within‐site variation and variation in habitat conditions, the occupied sites and sites in preferentially used substrates (i.e., pahoehoe and talus; Rodhouse et al., 2010; Jeffress et al., 2013; Rodhouse & Hovland, 2015) exhibited considerably more thermal buffering. These results add to the growing evidence (Hall et al., 2016; Henry et al., 2012; Millar & Westfall, 2010; Millar et al., 2014, 2016; Rodhouse et al., 2010; Varner & Dearing, 2014; Wilkening et al., 2011) that thermally stable subsurface microrefuges decoupled from regional climates are the mechanism by which American pikas persist in otherwise inhospitable conditions along the range periphery. Our study advances this line of inquiry beyond previous studies that have shown that subsurface microclimates are decoupled from aboveground climates (Hall et al., 2016; Henry et al., 2012; Millar et al., 2014, 2016; Shi et al., 2014; Varner & Dearing, 2014) and dispersal habitats (Millar et al., 2016) by demonstrating important differences in microclimates among used and unused sites and substrate types. Notably, our study is contextualized by an understanding of the species’ physiology, dispersal habits, and gene flow patterns provided for our study area by Castillo et al. (2016) as well as from concurrent multiyear monitoring efforts which consistently established that site occupancy probabilities vary substantially among these substrate types (Jeffress et al., 2013; Rodhouse & Hovland, 2015; Rodhouse et al., 2010). Furthermore, our monitoring has revealed striking geographic variation in rates of site occupancy turnover between the two study areas, with much greater variability and magnitude of turnover exhibited in the peripheral study area (Jeffress et al., 2013; Rodhouse & Hovland, 2015).

### 4.1 Among‐site variation

We tested three hypotheses about variation in subsurface microclimates between (1) occupied and unoccupied pika sites, (2) among sites with differing substrate complexities, and (3) substrates types; evidence was apparent in favor of the first and third hypotheses, but not the second. Measured microclimate differences between occupied and unoccupied sites were strongest for % stable, snow, and cold days; less substantial differences in summer heat metrics were apparent between occupied and unoccupied sites (Table 2), suggesting that either subsurface thermal buffering may be stronger during summer or that snow pack and cold stress exert a stronger influence on patterns of pika site occupancy than heat stress. Increasingly, evidence is mounting in favor of the latter that reduction in snow pack and winter cold stress may contribute as much or more to patterns of American pika distribution and extinction risk than summer heat stress (Beever et al., 2010; Erb et al., 2011; Guralnick et al., 2011; Schwalm et al., 2016). This is counterintuitive given the animal's physiological adaptations to colder climates (e.g., compact body size and metabolic rate; Smith & Weston, 1990) and intolerance of high temperatures, but it is plausible that cold stress contributes to higher rates of mortality (e.g., via brown fat thermogenesis), site turnover, and lower net site occupancy in cold sites observed in our study and by others (e.g., Kruezer & Huntly, 2003). Smith and Nagy (2015) also failed to find evidence that heat stress contributed to American pika occupancy dynamics over several decades. It is worth noting, however, that the apparent lack of influence of heat stress on site occupancy status may be confounded by the reliance on a threshold (e.g., 25.5°C in our study) that is presumably related to physiology (Smith, 1974) but has not been recently verified and could vary considerably among subspecies and ecotypes (e.g., adaptation to stress in peripheral populations; Mee & Moore, 2014). Wilkening et al. (2011) reported heat stress as the primary correlate between historically occupied talus patches with persisting populations and those with putatively extirpated populations. Hall et al. (2016) also found greater discrepancies between surface and subsurface temperatures in occupied pika sites in Wyoming. But Jeffress et al. (2013) and Schwalm et al. (2016) illustrated how the importance of heat stress in contemporary and projected future pika distribution patterns varied among different study areas as a result of local environmental idiosyncracies. The potential for geographic variation in physiological adaptations to heat (and cold) stress as an alternative explanation has not yet been investigated.

Surprisingly, our hypothesis that increasingly complex substrates would provide increasing levels of thermal stability was not supported (Table 2). Similar to the difficulties encountered in measuring crevice and ensuring consistent but meaningful sensor placement depth (also encountered by Wilkening et al., 2011 and Hall et al., 2016), developing a repeatable measure of substrate complexity and lava flow and talus bed depth is difficult (Jeffress et al., 2013). We used a subjective ranking scheme (ranked 1–3) of rock size and surface relief that reflects crevice abundance and trapped air space. This approach has been successfully used to differentiate pika occupancy probabilities among lava flow sites (Rodhouse et al., 2010; Shinderman, 2015). Millar and Westfall (2010) showed a strong association between patterns of pika site occurrence and structurally unique talus (rock‐ice features). Shi et al. (2014) has also hypothesized that rock structure variables such as size and layering would affect thermal buffering. Clearly, further investigation is warranted to more fully describe the role of substrate structural complexity in creating subsurface microrefuges, and for pika distribution patterns more generally. Advancements in remote sensing applications and other tools to describe substrate complexity are needed. For example, Schwalm et al. (2016) has applied high‐resolution digital elevation models generated from laser altimetry (LiDAR) to models of American pika distributions.

Our hypothesis that microclimate thermal stability would differ between a'a and pahoehoe lava substrates was well supported, as sensors located in a'a recorded significantly higher percentages of cold days in winter, and fewer stable days when compared with pahoehoe. Although these differences in temperatures were expected given the findings of Rodhouse et al. (2010), the exact mechanism(s) are unclear, and limited literature exists comparing the two lava substrates. Differences in substrate depth, density, and surface structural complexity likely influence buffering capability, although until more thorough physical descriptions of these substrates are made, we can only speculate. Notably, pikas persist in other low‐elevation lava environments, including in Lava Beds National Monument, California, USA (Jeffress et al., 2013; Ray et al., 2016), and in Newberry National Volcanic Monument, Oregon, USA (Shinderman, 2015), but it has not been established how these lava substrates compare, geologically, to those in Craters of the Moon. Studies of microclimate characteristics similar to ours conducted in these and in other low‐elevation peripheral environments (e.g., Beever et al., 2008) throughout the species’ range are necessary to verify our findings and enable the identification of potential microrefugia using broader site characteristics (e.g., via land cover mapping, LiDAR, and climate grids) and to enhance the value of current management tools (e.g., geologic maps, species distribution models).

Elevation is often used as a surrogate for temperature due to the adiabatic cooling rate associated with elevation gain (Körner, 2007) and has been routinely used to predict pika site occupancy patterns (e.g., Rodhouse et al., 2010). However, in our study, the influence of elevation on subsurface temperature patterns was insignificant, providing further evidence of the thermal buffering and climate decoupling capabilities of lava and talus substrates. Counter to the adiabatic cooling rate, positive subsurface temperature–elevation relationships have actually been recorded in warm seasons along elevational gradients in the eastern Sierra Nevada by Millar and Westfall (2010) and Millar et al. (2014, and attributed to cold‐air drainage. This underscores the importance of collecting climate data at biologically appropriate scales which capture temperatures actually experienced by the organism of study (Körner, 2007). Interestingly, topography had a greater influence on temperature than elevation, as steeper, north‐facing slopes consistently experienced a greater percentage of cold days than steep south‐facing slopes (Table 2). Other studies have shown steep north‐facing slopes to have a positive influence on pika site occupancy probabilities (Jeffress et al., 2013) and gene flow (Castillo et al., 2016), but it remains unclear as to why the strength of influence from topographic orientation was so much greater than that of elevation.

### 4.2 Within‐site variation

We found noteworthy discrepancies when comparing within‐site temperatures, revealing a high level of thermal heterogeneity within pika subsurface microrefuge habitats. Although previous studies of pika microrefuge habitat have also reported thermal heterogeneity on patch scales much larger than our 12‐m‐radius site occupancy plots (Erb et al., 2014; Henry et al., 2012; Varner & Dearing, 2014), these and other studies (Hall et al., 2016; Millar et al., 2016) have focused on subsurface decoupling from aboveground temperatures rather than on subsurface variation among surveyed sites, as we have performed here. Although we found statistically significant and biologically meaningful differences among‐site microclimates in spite of this heterogeneity, our findings indicate that a single sensor is unlikely to provide a representative description of microclimate, even at very fine scales. Our 12‐m‐radius plot sites (452 m^2^; see Jeffress et al., 2011, 2013 for details) approximate the size of individual pika territories (Smith & Weston, 1990; Varner et al., 2016). Therefore, the mechanisms influencing rates of site occupancy and turnover at territorial scales are likely to be very fine‐grained (e.g., crevice‐scale; Millar et al., 2016). Future studies should consider deploying ≥2 sensors, particularly for thermally sensitive species like the American pika. Furthermore, we note that within‐site replication would provide added analytical flexibility, for example for developing hierarchical (mixed‐effects or multilevel) models that can more explicitly model within‐ and among‐site variation. As our attempt to do so was unsuccessful with only two replicates per site (see the discrepancies reported in Table 1), we suggest as many as five sensors per site may be needed in future studies with designs similar to ours in order to achieve satisfactory multilevel modeling flexibility (Gelman & Hill, 2007).

The pattern of lower average within‐site discrepancy in Craters of the Moon compared to Crater Lake was intriguing and counterintuitive, given the higher thermal stability observed in Crater Lake subsurface microsites. We expected the deep and persistent snowpack at Crater Lake to reduce within‐site variation. However, Crater Lake has much higher topographic heterogeneity than the relatively flat lava flow plain in Craters of the Moon, which may explain some of the discrepancy. Millar et al. (2014) described the complex influences acting on montane talus and boulder‐field thermal characteristics (e.g., low vs. high talus positions); we would assume many of these same influences are relevant for lava flow environments as well, but this has not been described.

We suspect differences in substrate depths between the two parks is another likely explanation for between‐park differences in average temperature discrepancy. The lava flow depths at Craters of the Moon is reportedly >10 m thick (Kuntz et al., 2007) and potentially much deeper than Crater Lake talus. Although we placed sensors at comparable crevice depths in both parks, and accounted for depth in models, accurately measuring crevice depth is difficult (Hall et al., 2016; Jeffress et al., 2013; Wilkening et al., 2011) and evidently not the best reflection of potential substrate depth and subsurface airflow (Millar et al., 2014). Adding to this puzzle was the finding that high‐discrepancy sites were not always both hotter and colder, as might be expected if topographic position (e.g., aspect) was the primary influence. Rather, some microsites were simply colder than the neighboring microsite, or warmer, but not both, adding evidence that subsurface airflow may be the mechanism at work, similar to the dynamics of intratalus negative anomalies described in some montane talus systems (Millar & Westfall, 2010). Anecdotally, we have often felt cool breezes emerging from lava flows in Craters of the Moon, more so in pahoehoe than in a'a; these breezes have been described as a characteristic of negative anomalies in certain kinds of talus substrates (Millar & Westfall, 2010). Given the potential depths and internal structural complexities of lava flows at Craters of the Moon and in other low‐elevation lava flow environments with persistent but range‐peripheral pika populations (Jeffress et al., 2013; Shinderman, 2015), air‐flow‐driven thermal anomalies may be strong and highly influential mechanisms driving the kinds of microclimate differences described by our study. The insights from this study as well as from recent work by Millar et al. (2016) and Hall et al. (2016) make it increasingly evident that these anomalous subsurface microrefuges are the key mechanism for understanding and ultimately predicting more accurately American pika peripheral population persistence. Microhabitat‐scale processes are likely to be important for many other species experiencing climate change‐induced range contractions, especially along range peripheries; however, the fine scales, structural complexity, and spatial heterogeneity of these processes present a tremendous challenge to doing so.

## Conflict of Interest

None declared.

## Endnote

1 Available on‐line at http://www.onsetcomp.com/products/data-loggers/u22-001
